# Ultrasonographic changes in lower extremity tendon thickness after stroke rehabilitation and their associations with balance and functional outcomes

**DOI:** 10.3389/fneur.2026.1773636

**Published:** 2026-02-17

**Authors:** Tugba Alisik, Ebubekir Demir

**Affiliations:** Department of Physical Medicine and Rehabilitation, Faculty of Medicine, Bolu Abant İzzet Baysal University, Bolu, Türkiye

**Keywords:** hemiplegia, lower extremity, postural balance, stroke rehabilitation, tendons ultrasonography

## Abstract

**Introduction:**

Peripheral musculoskeletal structures may undergo secondary changes after stroke, but tendon-specific adaptations and their relationship with functional recovery are not well defined. This study examined lower extremity tendon thickness in patients with post-stroke hemiplegia before and after a four-week inpatient rehabilitation program and explored associations between tendon thickness and clinical improvement.

**Methods:**

In this prospective observational study, 45 patients with post-stroke hemiplegia completed a four-week rehabilitation program. Quadriceps, patellar, Achilles tendon and plantar fascia thicknesses were measured bilaterally at baseline and post-treatment using ultrasonography. Clinical assessments included the Berg Balance Scale (BBS), Functional Ambulation Classification (FAC), Barthel Index, Brunnstrom stages and Modified Ashworth Scale (MAS). Fifteen healthy volunteers served as controls (single assessment). Continuous variables are presented as mean ± SD when approximately normally distributed and as median (IQR) otherwise; *p*-values were adjusted for multiplicity in secondary analyses as specified.

**Results:**

Paretic-side quadriceps tendon thickness (primary outcome) increased from 5.94 ± 0.96 to 6.48 ± 0.95 mm (*p* < 0.001), with 21/45 (46.7%) exceeding minimal detectable change with a 95% confidence interval (MDC_95_). Baseline paretic-side quadriceps thickness was lower than controls (p_adj = 0.048) but did not differ post-treatment (p_adj > 0.99). Patellar and Achilles tendons and plantar fascia also showed consistent bilateral increases (all *p* < 0.001). Balance and functional outcomes improved over the period (BBS *Δ*: 6 [4–9]; FAC improved by ≥1 level in 27/45 [60.0%]; Barthel improved with median paired *Δ*: 0 [0–5]; all *p* < 0.001). Changes in quadriceps tendon thickness showed modest positive associations with changes in BBS (both sides) and Barthel (non-paretic side).

**Discussion:**

Lower-extremity tendon morphology in post-stroke hemiplegia appeared dynamic over a 4-week inpatient rehabilitation period, with quadriceps tendon thickness broadly paralleling improvements in balance and functional independence. Larger, longer-term studies are needed to clarify clinical utility.

## 1 Introduction

Stroke remains one of the leading causes of long-term disability worldwide, and many survivors continue to experience impairments in motor control, balance, and gait despite structured rehabilitation programs (1–5). Although the primary neurological injury explains much of this functional limitation, accumulating evidence indicates that peripheral musculoskeletal structures also undergo secondary alterations after stroke (2, 6–10). Reduced loading of the paretic limb, spasticity, asymmetrical gait mechanics, and prolonged immobilization may contribute to adaptive or maladaptive changes in tendon and muscle morphology (3, 8, 10–16). Despite these observations, tendon-specific structural changes in stroke survivors—particularly at weight-bearing lower-limb sites—remain only partially characterized, with available data scattered across small, site-specific studies (3, 8, 10–16).

Musculoskeletal ultrasonography has become an accessible and reliable tool for evaluating tendon and muscle architecture in neurological and orthopedic conditions (15, 17–21). Prior research in stroke rehabilitation has largely focused on changes in muscle thickness, fascicle length, muscle stiffness, or spasticity-related adaptations (2, 7–9, 12, 17). In contrast, the number of studies directly assessing tendon morphology in post-stroke patients is limited, with most data focusing on the Achilles tendon or describing entheseal alterations such as changes in the plantar fascia (3, 11–15, 22). A systematic review of ultrasonographic muscle and tendon properties in the spastic lower leg after stroke highlighted major gaps in longitudinal data and called for prospective studies starting early after stroke (8). A subsequent Achilles tendon study likewise emphasized the need for longitudinal designs that link tendon morphology to functional outcomes and targeted rehabilitation interventions (3) furthermore, recent tendon-focused work suggests that chronic hemiparesis may lead to increased Achilles tendon thickness (3, 11) and altered tendon mechanical properties compared with healthy or contralateral limbs (8, 22), yet evidence for similar adaptations in other tendons remains sparse and is mostly limited to cross-sectional fascia studies in the foot and lower leg (8, 13–16, 22). Little is known about how these structural changes evolve over the course of rehabilitation (8, 22).

Understanding how tendon properties respond during rehabilitation is clinically relevant, because tendon stiffness, thickness, and mechanical behavior influence muscle–tendon unit function and ankle joint mechanics, which in turn may contribute to gait capacity, balance, and ultimately functional independence after stroke (2, 10, 12, 23–25). Tendon-level adaptations may also help explain inter-individual variability in motor recovery and treatment responsiveness. Therefore, the primary aim of this study was to examine the within-patient change in quadriceps tendon thickness over a 4-week inpatient rehabilitation period. Secondary aims were to (i) compare quadriceps tendon thickness with healthy controls at baseline and post-treatment as cross-sectional reference comparisons, (ii) describe changes in other lower-limb tendons/fascia (patellar and Achilles tendon thicknesses and plantar fascia thickness), and (iii) assess prespecified associations between changes in quadriceps tendon thickness and changes in balance and functional independence.

## 2 Materials and methods

### 2.1 Study design and participants

This prospective observational study included patients with post-stroke hemiplegia who were admitted to the inpatient rehabilitation unit of Physical Medicine and Rehabilitation Clinic of İzzet Baysal Education and Research Hospital. Tendon and fascia thicknesses and clinical scales were evaluated at admission and re-evaluated after a 4-week rehabilitation program (20 sessions, 1 h/day). For each hemiplegic participant, ultrasound measurements were labeled as paretic (hemiplegic limb) and non-paretic (contralateral limb), and analyses were conducted using this paretic vs. non-paretic framework.

Adult patients with a first-ever ischemic or hemorrhagic stroke and hemiplegia were included. Exclusion criteria comprised: (1) history of peripheral neuropathy, diabetes-related polyneuropathy, or lower-limb surgery; (2) severe lower-limb contracture, deformity, or open wound preventing ultrasonographic assessment; (3) cognitive impairment interfering with cooperation; and (4) participation in other experimental therapies during the study period. Fifteen age- and sex-matched healthy volunteers served as controls.

### 2.2 Ethical approval

The study protocol was approved by the Bolu Abant İzzet Baysal University Non-Interventional Clinical Research Ethics Committee (decision no.: 2024/330) and conducted in accordance with the Declaration of Helsinki. Written informed consent was obtained from all participants.

### 2.3 Rehabilitation protocol

All patients received a standardized inpatient stroke rehabilitation program delivered 5 days/week for 4 weeks (20 sessions; ~60 min/session) by the same physiotherapy team. The program typically included: (i) lower-extremity range-of-motion and stretching; (ii) progressive strengthening of major lower-limb muscle groups (with resistance increased according to patient tolerance); (iii) balance training (static and dynamic postural control, weight shifting, and sit-to-stand practice); (iv) gait training (over ground walking practice with task-specific cueing and, when required, assistive devices); and (v) occupational therapy focused on activities of daily living. Functional electrical stimulation (FES) and ankle–foot orthoses (AFO)/assistive devices were prescribed when clinically indicated.

### 2.4 Clinical assessments

Clinical evaluations included the Brunnstrom Recovery Stages (26) [upper extremity (UE), hand, and lower extremity (LE)], Modified Ashworth Scales (MAS) (27) (hip, knee, foot), Functional Ambulation Classification (FAC) (28), Berg Balance Scale (BBS) (29, 30), and Barthel Index (31, 32). Each assessment was performed at baseline and after completion of the rehabilitation program by the same experienced physiatrist blinded to the ultrasonographic results.

### 2.5 Ultrasonographic measurements

Tendon thicknesses of the quadriceps, patellar, Achilles tendon, and plantar fascia were measured bilaterally using a high-resolution ultrasound system equipped with a 7–12 MHz linear transducer. All ultrasound procedures were performed using a V8 machine (Samsung Medison, Seoul, Korea) by a single physiatrist with >5 years of musculoskeletal ultrasound experience. Participants were examined in standardized relaxed positions, and the probe was kept perpendicular to the fiber orientation with minimal pressure to reduce anisotropy and compression artifacts.

Ultrasonographic evaluations of the quadriceps, patellar, and Achilles tendons, along with the plantar fascia, were conducted using a standardized protocol. For the quadriceps and patellar tendons, patients were positioned supine with the knee in 30° of flexion; measurements were taken 15 mm proximal to the superior pole of the patella and 15 mm distal to the inferior pole, respectively. Achilles tendon thickness was obtained in the prone position with the ankle in a neutral (90°) position over the edge of the table, at a point 2 cm proximal to the superior border of the calcaneal tuberosity. For the plantar fascia, measurements were recorded in the prone position with the hallux in passive dorsiflexion, targeting the origin of the fascia at the inferior aspect of the calcaneal tuberosity. All assessments were performed in the longitudinal plane along the midline, with the anteroposterior diameter recorded between the hyperechoic boundaries. Following the initial recording, the transducer was briefly detached from the skin and the measurement sequence was repeated for a second recording. The final values represent the mean of two consecutive measurements.

Intra-observer reliability was evaluated using repeated measurements obtained at the same session from all participants (*n* = 45). Reliability was quantified using a two-way mixed-effects model with absolute agreement for average measurements [ICC (3, 2)]. For each tendon/fascia and side, we report ICC with 95% confidence intervals, the standard error of measurement (SEM), and the minimal detectable change at the 95% confidence level (MDC_95_) (33). SEM was calculated as SEM = SD × √(1 − ICC) (where SD is the SD of baseline averaged thickness values (mean of two consecutive measurements) in the reliability dataset), and MDC_95_ as MDC95 = SEM × 1.96 × √2. Participants were classified as “responders” when the absolute pre–post change in thickness exceeded the site- and side-specific MDC_95_.

### 2.6 Statistical analysis

Statistical analyses were performed using JASP (version 0.95.4). Normality was assessed using the Shapiro–Wilk test and visual inspection of histograms. Continuous variables are presented as mean ± SD when approximately normally distributed and as median (inter quartile IQR) otherwise; categorical variables are presented as *n* (%).

Pre–post changes in BBS, Barthel Index, FAC, Brunnstrom stages (UE, hand, LE), and MAS were also evaluated using the Wilcoxon signed-rank test due to the ordinal nature of several scales or the non-normal distribution of scores. To improve interpretability of ordinal outcomes beyond median change, we additionally performed shift analyses summarizing the number (%) of participants who improved by ≥1 category, remained unchanged, or worsened (and step-change distributions where relevant). For MAS, baseline distributions and shift patterns were used to characterize potential floor effects. To support clinical interpretability, we additionally summarized the proportion of participants exceeding published minimally clinically important difference (MCID) thresholds for BBS and Barthel Index (34, 35). MCID-based responder proportions were reported descriptively.

Baseline-to-post-treatment changes in tendon thickness over the 4-week inpatient rehabilitation period were evaluated within patients. For the paretic-side quadriceps tendon, the baseline–post-treatment change was tested using a paired-samples t-test. The effect size was reported as Cohen’s dz. with 95% (confidence intervals) CIs. As a sensitivity analysis, the change was also examined using the Wilcoxon signed-rank test, yielding the same direction and statistical significance.

Cross-sectional reference comparisons with healthy controls were performed separately for the paretic and non-paretic sides in patients. For controls, right and left quadriceps tendon thickness values were averaged to obtain a single reference value per participant, which was then used for comparisons. These reference comparisons were conducted at baseline and post-treatment.

Between-group (patient vs. control) comparisons at each time point were performed using independent-samples t-tests. Between-group effect size was reported as Cohen’s d with 95% CIs. To control multiplicity for side-specific within-patient comparisons, Holm-adjusted *p*-values were calculated separately within each tendon/fascia, adjusting across the two side-specific tests [paretic (P) and non-paretic (NP)].

Correlation analyses were prespecified to test associations between changes in quadriceps tendon thickness (paretic and non-paretic sides) and changes in BBS and Barthel Index (four tests in total). Spearman’s rho is reported with 95% confidence intervals based on 1,000 bootstrap replicates. To address multiplicity across these four prespecified tests, *p*-values were adjusted using the Benjamini–Hochberg false discovery rate (FDR) procedure and q-values are reported.

Reliability metrics (ICC with 95% CIs, SEM, and MDC_95_) were reported for each tendon/fascia and side; the proportion exceeding MDC_95_ (“responders”) is provided to support interpretation of change beyond measurement error.

All analyses were two-tailed with *p* < 0.05 considered significant.

## 3 Results

Forty-five patients and fifteen controls were included. Median age was 64 (60–67) years in patients and 60 (55.5–64.5) years in controls (*p* = 0.185); sex distribution and anthropometrics were comparable between groups (*p* > 0.05 for all) (Table 1). Baseline stroke-related characteristics (including time since stroke, stroke type/side, and baseline Brunnstrom stages, FAC, BBS, Barthel Index, and MAS distributions) are presented in Table 1.

**Table 1:** Baseline demographic and clinical features of patients with post-stroke hemiplegia and healthy controls.

Variable	Category	Patient	Control	*p*-value
Age, years		64 (60 to 67)	60 (55.5 to 64.5)	0.185
Female sex		17 (37.8%)	8 (53.3%)	0.290
Height, cm		167.1 ± 7.0	167.3 ± 9.0	0.922
Weight, kg		80 (70 to 86)	75 (67.5 to 83)	0.189
Body mass index, kg/m^2^		27 (24 to 31)	27 (25 to 28)	0.829
Time since stroke, months		14 (5 to 20)		
Stroke type	Ischemic	32 (71.1%)		
	Hemorrhagic	13 (28.9%)		
Hemiplegic side	Right	25 (55.6%)		
	Left	20 (44.4%)		
Hypertension		36 (80%)		
Diabetes Mellitus		15 (33.3%)		
Coronary artery diseases		11 (24.4%)		
Smoking		8 (17.8%)		
Brunnstrom UE		4 (3 to 5)		
Brunnstrom Hand		4 (3 to 5)		
Brunnstrom LE		4 (4 to 5)		
Functional Ambulation Categories		3 (2 to 4)		
Berg Balance Scale		36 (27 to 44)		
Barthel Index		70 (50 to 85)		
MAS Hip	0	42 (93.3%)		
	1	1 (2.2%)		
	2	2 (4.4%)		
MAS Knee	0	42 (93.3%)		
	1	2 (4.4%)		
	2	1 (2.2%)		
MAS Foot	0	35 (77.8%)		
	1	2 (4.4%)		
	2	5 (11.1%)		
	3	3 (6.7%)		

Values are median (Q1–Q3), mean ± SD, or n (%). Between-group comparisons used Student’s *t*-test, Mann–Whitney U test, and Pearson’s chi-square test, as appropriate. MAS: Modified Ashworth Scale; UE: upper extremity; LE: lower extremity.

Ordinal outcomes were examined using shift analyses (Table 2; Supplementary Table S1). Brunnstrom stages showed predominantly favorable transitions with no worsening: Brunnstrom UE improved by ≥1 stage in 19/45 (42.2%), Brunnstrom LE in 15/45 (33.3%), and Brunnstrom Hand in 6/45 (13.3%), with the remainder unchanged (Table 2). FAC improved by ≥1 level in 27/45 (60.0%), while 18/45 (40.0%) remained unchanged (Table 2). In contrast, MAS demonstrated a marked floor effect—particularly at the hip and knee—where most participants were already graded 0 at baseline and remained unchanged (43/45, 95.6% for both); similarly, 35 (77.7%) participants were graded 0 at baseline for foot MAS, and overall 38/45 (84.4%) remained unchanged, with no participants worsening in any MAS domain. The full transition patterns by baseline category and post-treatment category are provided in Supplementary Table S1.

**Table 2:** Shift analysis of ordinal outcomes (Brunnstrom stages, FAC, and MAS) from baseline to post-treatment.

Outcome	Improved n/N (%)	Unchanged n/N (%)	Worsened n/N (%)	+1 step n/N (%)	+2 steps n/N (%)
Brunnstrom UE	19/45 (42.2)	26/45 (57.8)	0/45 (0.0)	19/45 (42.2)	0/45 (0.0)
Brunnstrom LE	15/45 (33.3)	30/45 (66.7)	0/45 (0.0)	14/45 (31.1)	1/45 (2.2)
Brunnstrom Hand	6/45 (13.3)	39/45 (86.7)	0/45 (0.0)	6/45 (13.3)	0/45 (0.0)
FAC	27/45 (60.0)	18/45 (40.0)	0/45 (0.0)	26/45 (57.8)	1/45 (2.2)
MAS Hip	2/45 (4.4)	43/45 (95.6)	0/45 (0.0)	2/45 (4.4)	0/45 (0.0)
MAS Knee	2/45 (4.4)	43/45 (95.6)	0/45 (0.0)	2/45 (4.4)	0/45 (0.0)
MAS Foot	7/45 (15.6)	38/45 (84.4)	0/45 (0.0)	7/45 (15.6)	0/45 (0.0)

Improved indicates a favorable shift (Brunnstrom/FAC: increase by ≥1 level; MAS: decrease by ≥1 grade). Values are n/N (%). FAC: Functional Ambulation Classification; MAS: Modified Ashworth Scale; UE: upper extremity; LE: lower extremity.

The BBS increased from 36 (27–44) at baseline to 42 (33–51) post-treatment (*Δ*: 6 [4–9], *p* < 0.001). Using a published MCID threshold of ≥5 points for the BBS, 29/45 (64.4%) participants achieved a change exceeding the MCID (34). The Barthel Index increased from 70 (50–85) at baseline to 75 (60–90) post-treatment (Δ: 0 [0–5], *p* < 0.001). Based on published MCID approaches for the Barthel Index in the stroke literature (including an estimate of 1.85 points on the 20-point BI, corresponding to approximately 9.25 points on a 0–100 scale), 10/45 (22.2%) participants exceeded the MCID criterion (35).

Measurement reliability and error estimates (ICC with 95% CIs, SEM, and MDC_95_) for each tendon/fascia and side are presented in Supplementary Table S2. For the quadriceps tendon, MDC_95_ was 0.50 mm on the paretic side and 0.45 mm on the non-paretic side.

For the primary outcome, paretic-side quadriceps tendon thickness increased from 5.94 ± 0.96 mm at baseline to 6.48 ± 0.95 mm post-treatment (*p* < 0.001), with a median paired change of 0.4 (0.3–0.8) mm (Table 3). The effect size was large (Cohen’s dz. = 1.358, 95% CI: 0.947–1.761), and 21/45 participants (46.7%) exceeded the MDC_95_ threshold on the paretic side. Quadriceps tendon thickness on the non-paretic side also increased (6.09 ± 0.83 to 6.54 ± 0.77 mm; *p* < 0.001; median *Δ* 0.3 mm [0.2–0.6]; dz. = 1.148, 95% CI: 0.766–1.521), with 17/45 (37.8%) exceeding MDC95 (Table 3). Secondary analyses showed statistically significant increases across the other sites on both sides (all *p* < 0.001). In sensitivity analyses, all pre–post comparisons remained statistically significant when re-checked using the Wilcoxon signed-rank test (all *p* < 0.001), and the direction of effects was unchanged.

**Table 3:** Within-patient changes in lower-limb tendon and plantar fascia thickness over the 4-week inpatient rehabilitation period.

Tendon/Fascia	Side	Baseline	Post-Tx	Delta change	*p*	Effect size (95% CI)	Responder *n* (%)
Quadriceps	P	5.94 ± 0.96	6.48 ± 0.95	0.4 (0.3–0.8)	< 0.001	1.358 (0.947–1.761)	21 (46.7%)
	NP	6.09 ± 0.83	6.54 ± 0.77	0.3 (0.2–0.6)	< 0.001	1.148 (0.766–1.521)	17 (37.8%)
Patellar	P	3.15 ± 0.4	3.35 ± 0.36	0.2 (0.1–0.3)	< 0.001	1.273 (0.874–1.663)	13 (28.9%)
	NP	3.23 ± 0.47	3.4 ± 0.41	0.1 (0–0.2)	< 0.001	0.827 (0.484–1.163)	11 (24.4%)
Achilles	P	4.38 ± 0.76	4.63 ± 0.7	0.2 (0.1–0.4)	< 0.001	1.021 (0.656–1.379)	17 (37.8%)
	NP	4.38 ± 0.7	4.6 ± 0.65	0.2 (0–0.3)	< 0.001	0.809 (0.468–1.143)	13 (28.9%)
Plantar fascia	P	2.80 ± 0.44	2.95 ± 0.42	0.1 (0–0.2)	< 0.001	0.915 (0.563–1.260)	10 (22.2%)
	NP	2.80 ± 0.45	2.94 ± 0.44	0.1 (0–0.2)	< 0.001	0.869 (0.521–1.209)	6 (13.3%)

Values are mean ±S D. Post-Tx: Post treatment, P = paretic side; NP = non-paretic side. Delta change is presented as median (IQR) of paired differences. Primary and secondary within-patient pre–post comparisons were evaluated using paired-samples t-tests, and effect size is reported as Cohen’s dz with 95% confidence intervals. “Responder” indicates the number (%) of participants whose absolute change exceeded the site- and side-specific MDC95. Sensitivity analysis: All pre–post comparisons were re-checked using the Wilcoxon signed-rank test; all remained statistically significant (*p* < 0.001) and the direction of effects was unchanged.

Cross-sectional comparisons with healthy controls at baseline and post-treatment are summarized in Table 4 (Holm-adjusted *p*-values reported separately within each tendon/fascia for the two side-specific comparisons, and separately for baseline and post-treatment). At baseline, quadriceps tendon thickness on the paretic side was lower in patients than controls (5.94 ± 0.96 vs. 6.58 ± 0.87 mm; *p* = 0.024; p_adj = 0.048; Cohen’s d = −0.689, 95% CI: −1.284 to −0.089), whereas the non-paretic side did not remain significant after adjustment (*p* = 0.054; p_adj = 0.108). At post-treatment, quadriceps thickness did not differ from controls on either side (both p_adj > 0.999). Post-treatment patellar and Achilles tendon thicknesses were higher in patients than controls on both sides (all p_adj ≤ 0.010). All other baseline and post-treatment patient–control comparisons were not statistically significant after Holm adjustment (Table 4).

**Table 4:** Baseline and post-treatment cross-sectional comparisons of lower-limb tendon and plantar fascia thickness in post-stroke hemiplegia versus healthy controls.

Tendon/Fascia	Side	Baseline	Post-Tx	Control	Baseline vs. control			Post-Tx vs. control		
					*p*	p_adj	Cohen’s d	*p*	p_adj	Cohen’s d (95% CI)
Quadriceps	P	5.94 ± 0.96	6.48 ± 0.95	6.58 ± 0.87	0.024	0.048	−0.689 (−1.284 to −0.089)	0.717	>0.999	−0.109 (−0.693 to 0.476)
	NP	6.09 ± 0.83	6.54 ± 0.77	6.58 ± 0.87	0.054	0.108	−0.588 (−1.179 to 0.009)	0.848	>0.999	−0.057 (−0.642 to 0.527)
Patellar	P	3.15 ± 0.4	3.35 ± 0.36	3.04 ± 0.36	0.316	0.632	0.302 (−0.286 to 0.887)	0.005	0.010	0.878 (0.269 to 1.480)
	NP	3.23 ± 0.47	3.40 ± 0.41	3.04 ± 0.36	0.160	0.320	0.425 (−0.166 to 1.012)	0.003	0.006	0.917 (0.306 to 1.521)
Achilles	P	4.38 ± 0.76	4.63 ± 0.70	4.04 ± 0.59	0.117	0.234	0.475 (−0.118 to 1.063)	0.005	0.010	0.875 (0.266 to 1.477)
	NP	4.38 ± 0.70	4.60 ± 0.65	4.04 ± 0.59	0.093	0.186	0.508 (−0.085 to 1.098)	0.004	0.008	0.888 (0.278 to 1.491)
Plantar fascia	P	2.80 ± 0.44	2.95 ± 0.42	2.70 ± 0.51	0.479	0.958	0.212 (−0.374 to 0.797)	0.062	0.124	0.566 (−0.029 to 1.157)
	NP	2.80 ± 0.45	2.94 ± 0.44	2.70 ± 0.51	0.476	0.952	0.214 (−0.373 to 0.799)	0.072	0.144	0.546 (−0.049 to 1.136)

Values are mean ± SD. Post-Tx: Post treatment, P = paretic side; NP = non-paretic side; CI: confidence intervals. “Control” indicates the healthy control group (single cross-sectional assessment). For the control group, thickness values represent the mean of the right and left sides (i.e., a bilateral average). This approach was used to provide a single normative reference value per tendon/fascia for the control group. Pre–control and post–control comparisons are cross-sectional reference comparisons (controls were not followed longitudinally). For multiplicity control, Holm-adjusted p-values (p_adj) were calculated separately for each tendon/fascia and separately for pre–control and post–control comparisons, adjusting across the two side-specific tests (P and NP) within each tendon.

In prespecified correlation analyses (four tests; FDR-adjusted q-values reported), changes in quadriceps tendon thickness were modestly associated with improvements in balance and functional independence (Table 5). ΔQuadriceps was positively correlated with ΔBBS on both sides (paretic: rho = 0.346, 95% CI: 0.037 to 0.622, *p* = 0.020, q = 0.037; non-paretic: rho = 0.365, 95% CI: 0.100 to 0.585, *p* = 0.014, q = 0.037). ΔQuadriceps_NP also showed a modest positive association with ΔBarthel (rho = 0.329, 95% CI: 0.015 to 0.580, *p* = 0.028, q = 0.037), whereas ΔQuadriceps_P was not associated with ΔBarthel (rho = 0.131, 95% CI: −0.205 to 0.422, *p* = 0.392, q = 0.392). The full correlation heatmap was considered exploratory and is provided in the Supplementary Figure S1.

**Table 5:** Correlations between changes in quadriceps tendon thickness and changes in balance and functional independence.

Variable pair	Spearman’s rho	95% CI (bootstrap)	p	q (FDR-BH)
ΔQuadriceps_P vs. *Δ*BBS	0.346	0.037 to 0.622	0.020	**0.037**
ΔQuadriceps_NP vs. ΔBBS	0.365	0.100 to 0.585	0.014	**0.037**
ΔQuadriceps_P vs. ΔBarthel	0.131	−0.205 to 0.422	0.392	0.392
ΔQuadriceps_NP vs. ΔBarthel	0.329	0.015 to 0.580	0.028	**0.037**

P = paretic side; NP = non-paretic side; BBS = Berg Balance Scale. Δ denotes within-patient change (post–baseline). Spearman’s rho is reported with 95% confidence intervals based on 1,000 bootstrap replicates. q denotes Benjamini–Hochberg FDR-adjusted p-values across the four prespecified tests.

## 4 Discussion

This study investigated ultrasound-based thickness changes in major lower-limb tendons and plantar fascia structures in individuals with post-stroke hemiplegia and explored how these changes relate to functional recovery over a four-week inpatient rehabilitation period. The primary finding was a within-patient increase in quadriceps tendon thickness over the 4-week inpatient period. Baseline patient–control differences were observed only on the paretic side, and post-treatment cross-sectional reference comparisons suggested no clear difference from controls; however, controls were not followed longitudinally. Over the same period, the patellar tendon, Achilles tendon, and plantar fascia thicknesses also demonstrated bilateral increases, although of smaller magnitude. In addition, quadriceps tendon thickness change showed modest associations with improvements in balance and functional independence, whereas other correlations were weak. Importantly, MDC_95_-based responder analyses indicated that a substantial subset of participants demonstrated Quadriceps tendon thickness changes exceeding measurement error, and MCID-based summaries suggested that many participants achieved clinically meaningful improvement in balance (BBS), whereas fewer met MCID-based criteria for functional independence (Barthel). Because we lacked a longitudinal control group or non-intervention comparator, the observed changes should be interpreted as time-related change over the inpatient period (potentially reflecting natural recovery, hospitalization-related mobilization/activity, and standard rehabilitation exposure), rather than as definitive rehabilitation effects. Taken together, these findings suggest that peripheral tendon thickness change may accompany functional gains during neurorehabilitation and may reflect adaptations related to changes in mechanical loading of the lower extremity.

Reduced baseline quadriceps tendon thickness is consistent with known mechanisms of post-stroke musculoskeletal deconditioning, including reduced paretic-extremity loading, asymmetrical gait patterns, diminished voluntary activation, and spasticity-driven disuse (1, 8, 21, 23). Previous work has described a range of post-stroke muscle architectural changes—including reduced muscle thickness, shorter fascicles, increased stiffness and impaired mechanical properties—in the quadriceps, tibialis anterior and gastrocnemius muscles (2, 3, 6, 7, 9). However, tendon-specific data remain limited (8). A systematic review of ultrasonographic muscle and tendon properties in the spastic lower leg reported evidence of altered tendon characteristics in stroke survivors, but emphasized that longitudinal studies starting early after stroke are still lacking (8). Our findings add to this limited literature by suggesting that quadriceps tendon morphology is already altered at baseline and may change over a short, structured inpatient rehabilitation period.

Over the 4-week inpatient period, we observed increases in thickness across all examined sites, with the quadriceps tendon demonstrating the largest changes. Tendons are biologically responsive to repetitive loading, and increases in cross-sectional thickness are generally interpreted as reflecting fibrillar reorganization, altered collagen turnover, and/or changes in water content (24, 25). Prior work focusing primarily on the Achilles tendon has reported post-stroke alterations in tendon morphology and mechanics, including increased thickness, reduced stiffness and Young’s modulus, and indications of disrupted collagen organization and abnormal mechanical behavior (3, 8, 11, 12, 22). The current results suggest that such thickness changes over the rehabilitation period is not restricted to posterior-chain tendons; the patellar tendon and plantar fascia also appear sensitive to the cumulative loading associated with strengthening, gait training and balance exercises. This pattern is consistent with studies demonstrating widespread entheseal and fascial abnormalities at the quadriceps, patellar, Achilles, and plantar fascia insertions in metabolic disease and in stroke cohorts (13–16, 18), and may reflect a similar tendency toward multi-site tendon and fascia involvement in stroke-related deconditioning. A key interpretive issue is whether the observed thickness changes exceed measurement error. Using repeated acquisitions with probe removal and repositioning, MDC_95_-based responder analyses showed that 46.7% of participants on the paretic side and 37.8% on the non-paretic side exceeded MDC_95_ for quadriceps thickness. Thus, while group-level changes were robust, individual-level changes were close to the limits of detectability in a subset, underscoring the importance of interpreting small absolute differences alongside SEM/MDC rather than relying on statistical significance alone.

The observed associations between quadriceps tendon thickness change and improvements in both balance (BBS) and activities of daily living (Barthel) may have functional relevance. The quadriceps plays a key role in knee control, weight acceptance, postural stability and sit-to-stand transitions-domains that are strongly represented in these clinical scales (1, 2, 10, 23). Increased tendon thickness might reflect improved neuromuscular engagement and more symmetrical loading patterns during rehabilitation, in line with evidence that muscle–tendon unit properties influence gait mechanics and functional performance (10, 12, 24, 25). Although effect sizes were modest, they are consistent with the notion that peripheral tendon adaptations may accompany central motor recovery and contribute to functional gains (3, 8, 10). Other site–outcome correlations are reported as exploratory in the Supplementary material. In addition, we contextualized functional improvements using published MCID thresholds. In our cohort, a substantial proportion of participants achieved clinically meaningful improvement in balance based on the published BBS MCID threshold (34). In contrast, fewer participants exceeded MCID-based criteria for the Barthel Index (35), which is consistent with the relatively smaller paired changes observed in functional independence over a short inpatient period.

Beyond the quadriceps tendon, our data also showed measurable but smaller changes in the patellar tendon, Achilles tendon and plantar fascia. At baseline, these structures did not exhibit the same degree of between-group difference as the quadriceps tendon, yet rehabilitation was associated with bilateral increases rather than a strictly paretic-extremity–specific response. In MDC_95_-based responder analyses, the proportion exceeding MDC_95_ was lower for these sites than for the quadriceps (e.g., ~24–29% for patellar, ~29–38% for Achilles, and ~13–22% for plantar fascia), supporting that individual-level change beyond measurement error was present but less frequent and more heterogeneous. This pattern may reflect a more global adjustment of the lower-extremity tendon–fascia complex to increased ambulatory loading and task practice, consistent with reports of structural alterations in the Achilles tendon and plantar fascia in stroke and other chronic loading conditions (3, 11, 12, 15, 18, 22). The absence of stronger correlations between changes in these structures and BBS or Barthel scores may indicate that our functional scales are less sensitive to plantar flexor- and foot-related contributions to gait and balance, or that remodeling in these regions contributes more to other domains, such as propulsion, endurance or pain, which were not specifically captured by the outcome measures used in this study (10, 12, 24, 25).

Several methodological strengths enhance the relevance of this work, including bilateral assessment of four major lower-extremity structures, the use of standardized ultrasonographic protocols, and the prospective design allowing short-term structural tracking. Integrating tendon thickness with clinical outcomes provides a more comprehensive view of potential peripheral contributions to stroke recovery. To reduce the risk of chance findings, we prespecified Quadriceps tendon thickness as the primary outcome, applied multiplicity control for side-specific secondary thickness comparisons, and limited correlation testing to a small prespecified set. Nevertheless, some limitations should be acknowledged. First, the observational pre–post design without a longitudinal control group or a non-intervention comparator limits causal inference; therefore, the observed changes should be interpreted as time-related change over the inpatient period, potentially reflecting natural recovery, hospitalization-related mobilization, and standard rehabilitation exposure. Second, we provide quantitative context for sample size using observed effects from this cohort. For the primary outcome, the paired effect size was large (Cohen’s dz. = 1.36, 95% CI: 0.95–1.76); under two-sided *α* = 0.05, a paired design would require approximately *n* ≈ 9 participants to detect dz. = 0.95 with 80% power, whereas with *n* = 45 the minimum detectable paired effect is approximately dz. ≈ 0.42 at 80% power, indicating that the primary comparison was well powered for moderate-to-large effects. In contrast, association analyses are more sample-demanding; with *n* = 45, power is ~80% only for correlations of approximately |*ρ*| ≥ 0.41, so smaller-to-moderate correlations (e.g., ρ ≈ 0.30–0.35) should be interpreted as preliminary. Third, while B-mode ultrasonography provides accessible information on gross morphology, it cannot characterize collagen organization or mechanical properties, which could be explored using elastography or MRI (17, 22, 36). Fourth, the rehabilitation program was standardized but tendon-specific loading dose was not quantified, limiting mechanistic interpretation. Fifth, time since stroke was heterogeneous, which may modify the relative contribution of spontaneous recovery versus therapy exposure. Sixth, some clinical outcomes are ordinal and may show floor/ceiling effects; in our cohort, MAS demonstrated a marked floor effect, particularly at the hip and knee, limiting sensitivity to detect change over a short interval. Finally, long-term follow-up was not available, and the durability of thickness changes remains unknown.

## 5 Conclusion

In conclusion, our findings suggest that tendon morphology in post-stroke hemiplegia is not static but can undergo measurable thickness change over a 4-week inpatient rehabilitation period. Within this cohort, quadriceps tendon thickness appeared particularly prominent, and paralleled modest gains in balance and functional independence. These observations raise the possibility that monitoring tendon adaptations could provide additional insight into peripheral contributions to motor recovery and, in larger and longer-term studies, might help refine individualized rehabilitation strategies.

## Data Availability

The raw data supporting the conclusions of this article will be made available by the authors, without undue reservation.

## References

[1] Niewolak K Antkiewicz J Piejko L Sobota G Maszczyk A Nawrat-Szołtysik A . Assessment of postural control and gait in patients with chronic stroke after treadmill perturbation-based training: a randomized clinical trial. J Clin Med. (2025) 14:6142. doi: 10.3390/jcm14176142, 40943902 PMC12429574

[2] Özbek İC Akgül Ö Tıkız C Ünlü Z Cerrahoğlu L. Muscle quality and structural changes in stroke patients: an ultrasonographic evaluation. Turk J Osteoporos. (2025) 18:67934. doi: 10.4274/tod.galenos.2025.67934

[3] Liang JN Bashford G Kulig K Ho KY. Achilles tendon morphology adaptations in chronic post-stroke hemiparesis: a comparative analysis with neurologically intact controls. Front Sports Act Living. (2024) 6:1498333. doi: 10.3389/fspor.2024.1498333, 39839548 PMC11745886

[4] Liu Y Jiang M Pan X Geng J. Effects of exercise on mobility, balance and gait in patients with the chronic stroke: a systematic review and Meta-analysis. Sci Rep. (2025) 15:24158. doi: 10.1038/s41598-025-09458-1, 40619517 PMC12230125

[5] Alisik T Alisik M Cogalgil S. The relationship between rehabilitation outcomes and extracellular thiol-disulphide and intracellular oxidized-reduced glutathione homeostasis in patients with subacute stroke. Neurol Asia. (2023) 28:1–15. doi: 10.54029/2023rye

[6] Körting C Schlippe M Petersson S Pennati GV Tarassova O Arndt A . In vivo muscle morphology comparison in post-stroke survivors using ultrasonography and diffusion tensor imaging. Sci Rep. (2019) 9:11836. doi: 10.1038/s41598-019-47968-x, 31413264 PMC6694129

[7] Kim JM Tay MRJ Rajeswaran DK Tham S-L Lui WL Kong KH. Changes in muscle architecture on ultrasound in patients early after stroke. NeuroRehabilitation. (2021) 49:565–72. doi: 10.3233/NRE-210257, 34806627

[8] Schillebeeckx F De Groef A De Beukelaer N Desloovere K Verheyden G Peers K. Muscle and tendon properties of the spastic lower leg after stroke defined by ultrasonography: a systematic review. Eur J Phys Rehabil Med. (2021) 57:495–510. doi: 10.23736/S1973-9087.20.06462-X, 33305547

[9] González-Buonomo J Alexander HP Ghuman J Malik A Yozbatiran N Francisco GE . Ultrasound assessment of spastic muscles in ambulatory chronic stroke survivors reveals function-dependent changes. J Rehabil Med. (2023) 55:3199. doi: 10.2340/jrm.v54.3199 36254740 PMC9880876

[10] Zhang L Van Wouwe T Yan S Wang R. Emg-constrained and ultrasound-based muscle-tendon parameter estimation in post-stroke subject. Gait Posture. (2022) 97:S295–6. doi: 10.1109/TBME.2024.3352556 38206783

[11] Liang JN Ho K-Y. Altered Achilles tendon morphology in individuals with chronic post-stroke hemiparesis: a case report. BMC Med Imaging. (2020) 20:34. doi: 10.1186/s12880-020-00431-0, 32245424 PMC7119093

[12] Freire B Dias CP Goulart NBA de Castro CD Becker J Gomes I . Achilles tendon morphology, plantar flexors torque and passive ankle stiffness in spastic hemiparetic stroke survivors. Clin Biomech. (2017) 41:72–6. doi: 10.1016/j.clinbiomech.2016.12.004 27992779

[13] Calvo-Lobo C Useros-Olmo AI Almazán-Polo J Martín-Sevilla M Romero-Morales C Sanz-Corbalán I . Quantitative ultrasound imaging pixel analysis of the intrinsic plantar muscle tissue between hemiparesis and contralateral feet in post-stroke patients. Int J Environ Res Public Health. (2018) 15:2519. doi: 10.3390/ijerph15112519, 30423860 PMC6265729

[14] Calvo-Lobo C Useros-Olmo AI Almazán-Polo J Becerro-de-Bengoa-Vallejo R Losa-Iglesias ME Palomo-López P . Rehabilitative ultrasound imaging of the bilateral intrinsic plantar muscles and fascia in post-stroke survivors with hemiparesis: a case-control study. Int J Med Sci. (2018) 15:907–14. doi: 10.7150/ijms.25836, 30008603 PMC6036101

[15] Şen S. Ultrasonographic measurement of plantar fascia thickness in patients with hemiplegia. J Contemp Med. (2023) 13:509–13. doi: 10.16899/jcm.1244249

[16] Choi J Do Y Lee H. Ultrasound imaging comparison of crural fascia thickness and muscle stiffness in stroke patients with spasticity. Diagnostics. (2024) 14:2606. doi: 10.3390/diagnostics14222606, 39594272 PMC11592608

[17] Roots J Trajano GS Fontanarosa D. Ultrasound Elastography in the assessment of post-stroke muscle stiffness: a systematic review. Insights Imaging. (2022) 13:67. doi: 10.1186/s13244-022-01191-x, 35380302 PMC8982789

[18] Okur SC Dogan YP Mert M Aksu O Burnaz O Caglar NS. Ultrasonographic evaluation of lower extremity Entheseal sites in diabetic patients using Glasgow ultrasound Enthesitis scoring system score. J Med Ultrasound. (2017) 25:150–6. doi: 10.1016/j.jmu.2017.03.011, 30065480 PMC6029304

[19] Cushman DM Carefoot A Corcoran B Vu L Fredericson M Fausett C . Prevalence of sonographic Achilles tendon, patellar tendon, and plantar fascia abnormalities in division I collegiate athletes from a variety of sports. Clin J Sport Med. (2024) 34:297–303. doi: 10.1097/jsm.0000000000001183, 37540559 PMC10838354

[20] Meyer NB Jacobson JA Kalia V Kim SM. Musculoskeletal ultrasound: athletic injuries of the lower extremity. Ultrasonography. (2018) 37:175–89. doi: 10.14366/usg.18013, 29794963 PMC6044222

[21] Liu J Pan H Bao Y Huang L Hu Y. The clinical utility of musculoskeletal ultrasonography in hemiplegic shoulder rehabilitation Poststroke. Front Rehabil Sci. (2025) 6:1576890. doi: 10.3389/fresc.2025.1576890, 40443485 PMC12119546

[22] Shao YH Peng Z Kong X Wang B Zhang H. Real-time ultrasound elastography evaluation of Achilles tendon properties in patients with mild hemiplegic stroke after rehabilitation training. J Ultrasound Med. (2019) 38:713–23. doi: 10.1002/jum.14755 30280400

[23] Li J Zhong D Ye J He M Liu X Zheng H . Rehabilitation for balance impairment in patients after stroke: a protocol of a systematic review and network Meta-analysis. BMJ Open. (2019) 9:e026844. doi: 10.1136/bmjopen-2018-026844, 31326927 PMC6661695

[24] Smith RE Shelton AD Sawicki GS Franz JR. The effects of Plantarflexor weakness and reduced tendon stiffness with aging on gait stability. PLoS One. (2024) 19:e0302021. doi: 10.1371/journal.pone.0302021, 38625839 PMC11020829

[25] Dong Z Moon Y Lee SK Yun HY Song J Xu J . Associations of lower limb muscle-tendon properties with dual-task gait variability: a cross-age study. Healthcare. (2025) 13:1375. doi: 10.3390/healthcare13121375, 40565402 PMC12192709

[26] Brunnstrom S. Motor testing procedures in hemiplegia: based on sequential recovery stages. Phys Ther. (1966) 46:357–75. doi: 10.1093/ptj/46.4.357, 5907254

[27] Bohannon RW Smith MB. Interrater reliability of a modified Ashworth scale of muscle spasticity. Phys Ther. (1987) 67:206–7. doi: 10.1093/ptj/67.2.206, 3809245

[28] Holden MK Gill KM Magliozzi MR Nathan J Piehl-Baker L. Clinical gait assessment in the neurologically impaired. Reliability and meaningfulness. Phys Ther. (1984) 64:35–40. doi: 10.1093/ptj/64.1.35, 6691052

[29] Berg KO Wood-Dauphinee SL Williams JI Maki B. Measuring balance in the elderly: validation of an instrument. Can J Public Health. (1992) 83:S7–S11. 1468055

[30] Şahin F Büyükavcı R Sağ S Doğu B Kuran B. Reliability and validity of the Turkish version of the Berg balance scale in patients with stroke. Türk Fiz Tıp Rehab Derg. (2013) 59:170–5. doi: 10.4274/tftr.02212

[31] Collin C Wade DT Davies S Horne V. The Barthel Adl index: a reliability study. Int Disabil Stud. (1988) 10:61–3. doi: 10.3109/09638288809164103, 3403500

[32] Kucukdeveci AA Yavuzer G Tennant A Suldur N Sonel B Arasil T. Adaptation of the modified Barthel index for use in physical medicine and rehabilitation in Turkey. Scand J Rehabil Med. (2000) 32:87–92. doi: 10.1080/16501977328792, 10853723

[33] Muckelt PE Warner MB Cheliotis-James T Muckelt R Hastermann M Schoenrock B . Protocol and reference values for minimal detectable change of Myotonpro and ultrasound imaging measurements of muscle and subcutaneous tissue. Sci Rep. (2022) 12:13654. doi: 10.1038/s41598-022-17507-2, 35953503 PMC9372175

[34] Tamura S Miyata K Kobayashi S Takeda R Iwamoto H. The minimal clinically important difference in Berg balance scale scores among patients with early subacute stroke: a multicenter, retrospective, observational study. Top Stroke Rehabil. (2022) 29:423–9. doi: 10.1080/10749357.2021.1943800 34169808

[35] Hsieh YW Wang CH Wu SC Chen PC Sheu CF Hsieh CL. Establishing the minimal clinically important difference of the Barthel index in stroke patients. Neurorehabil Neural Repair. (2007) 21:233–8. doi: 10.1177/1545968306294729, 17351082

[36] Lin TY Shen PC Chang KV Wu WT Özçakar L. Shoulder ultrasound imaging in the post-stroke population: a systematic review and Meta-analysis. J Rehabil Med. (2023) 55:jrm13432. doi: 10.2340/jrm.v55.13432, 37615388 PMC10461179

