# Supplementary material for: Ultrasonographic changes in lower extremity tendon thickness after stroke rehabilitation and their associations with balance and functional outcomes
Source: Front Neurol. 2026 Feb 17;17:1773636. doi: 10.3389/fneur.2026.1773636 (PMC12953124; doi:10.3389/fneur.2026.1773636)
Supplement: Supplementary file 1 [file Table_1.docx]

Supplementary Material

Supplementary Table S1. Shift (transition) analysis for ordinal clinical outcomes over the 4-week inpatient rehabilitation period

| Panel A. Brunnstrom stages and Functional Ambulation Classification (FAC): T0 → T1 shifts | | | | |
| --- | --- | --- | --- | --- |
| Stage/level shift | Brunnstrom UE | Brunnstrom LE | Brunnstrom Hand | FAC |
| 0→1 | N/A | N/A | N/A | 1/1 |
| 1→2 | 1/2 | - | 1/5 | 5/9 |
| 2→3 | 3/6 | 1/1 | 1/6 | 4/7 |
| 2→4 | - | - | - | 1/7 |
| 3→4 | 4/9 | 2/10 | 1/6 | 9/13 |
| 3→5 | - | 1/10 | - | - |
| 4→5 | 4/10 | 8/20 | 0/13 | 7/15 |
| 5→6 | 7/16 | 3/13 | 3/13 | N/A |
| Unchanged | 26/45 | 30/45 | 39/45 | 18/45 |
| *P value* | *<0.001* | *<0.001* | *0.020* | *<0.001* |
| Panel B. Modified Ashworth Scale (MAS): T0 → T1 shifts | | | | |
| Gade shift | MAS Hip | MAS Knee | MAS Foot |  |
| 0→1 | 0/42 | 0/42 | 0/35 |  |
| 1→0 | 1/1 | 1/2 | 2/2 |  |
| 2→1 | 1/2 | 1/1 | 3/5 |  |
| 3→2 | - | - | 2/3 |  |
| Unchanged | 43/45 | 43/45 | 38/45 |  |
| *P value* | *0.346* | *0.346* | *0.011* |  |

N/A: not available (Brunnstrom doesn’t have level 0 and FAC doesn’t have stage six). Values are shown as n/N, where N is the number of participants in the baseline (T0) category (row) and n is the number who moved to the specified post-treatment (T1) category. “–” indicates that no participants were observed in that transition. “N/A” indicates transitions that are not applicable because the scale does not include that category (Brunnstrom stages do not include level 0; FAC does not include level 6). Abbreviations: UE, upper extremity; LE, lower extremity; MAS, Modified Ashworth Scale; FAC, Functional Ambulation Classification; T0, baseline; T1, post-treatment.

Supplementary Table S2. Intraclass correlation coefficients (ICC), standard error of measurement (SEM), and minimal detectable change (MDC) values for ultrasound-measured tendon thicknesses on the paretic and non-paretic sides

| Tendon/fascia | Side | N | ICC(3,2) (95% CI) | SD | SEM | MDC95 |
| --- | --- | --- | --- | --- | --- | --- |
| Quadriceps | P | 45 | 0.965 (0.936-0.981) | 0.958 | 0.182 | 0.50 |
|  | NP | 45 | 0.961 (0.929-0.979) | 0.833 | 0.164 | 0.45 |
| Patellar | P | 45 | 0.927 (0.868-0.960) | 0.395 | 0.107 | 0.30 |
|  | NP | 45 | 0.963 (0.932-0.980) | 0.472 | 0.092 | 0.25 |
| Achilles | P | 45 | 0.979 (0.961-0.988) | 0.755 | 0.112 | 0.30 |
|  | NP | 45 | 0.977 (0.959-0.987) | 0.695 | 0.106 | 0.29 |
| Plantar fascia | P | 45 | 0.951 (0.911-0.973) | 0.437 | 0.097 | 0.27 |
|  | NP | 45 | 0.938 (0.887-0.966) | 0.447 | 0.111 | 0.31 |

P, paretic side, NP, non-paretic side, ICC, intraclass correlation coefficient; SD, standard deviation; SEM, standard error of measurement; MDC_95_, minimal detectable change at the 95% confidence level.

**
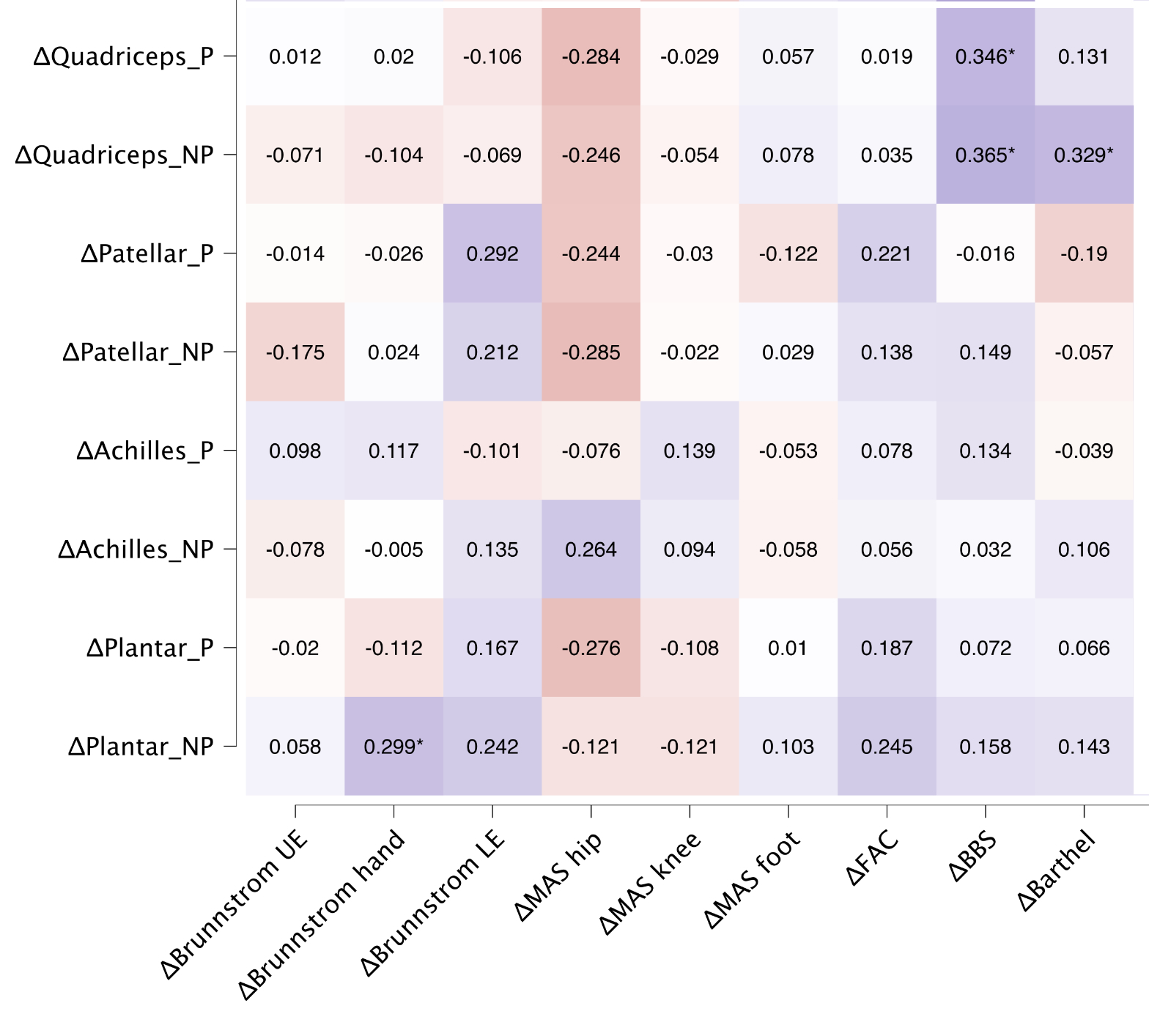
**

Supplementary Figure S1. Correlation between changes (Δ) in tendon thickness (quadriceps, patellar, Achilles, plantar; paretic (P) and non‑paretic (NP) sides) and changes in motor function, spasticity, balance, ambulation, and activities. Cells display Spearman’s correlation coefficients (rho). Asterisks denote statistical significance (p<0.05). UE: Upper extremity, LE: Lower extremity, MAS: Modified Ashworth Scale, FAC: Functional Ambulation Categories, BBS: Berg Balance Scale. ΔBrunnstrom UE/Hand/LE, ΔMAS Hip/Knee/Foot, ΔFAS, ΔBBS, and ΔBarthel refer to change from baseline to post‑treatment.
